# Lentiviral rescue of UMPS auxotrophy enables drug-free selection and stable vector expression

**DOI:** 10.1016/j.omta.2026.201761

**Published:** 2026-05-15

**Authors:** Henrike Steding, Nicole Dörpmund, Jessica Herbst, Martin Sauer, Tobias Maetzig

**Affiliations:** 1 Department of Pediatric Hematology and Oncology, Hannover Medical School, 30625 Hannover, Germany; 2 Institute of Experimental Hematology, Hannover Medical School, 30625 Hannover, Germany

**Keywords:** lentiviral gene transfer, vector silencing, transgene expression, auxotrophy, multiplexing, fluorescent genetic barcoding, UMPS

## Abstract

Retroviral gene transfer remains a cornerstone of biomedical research and gene therapy, yet its effectiveness is occasionally compromised by low transduction efficiency, transgene silencing, and the reduced fitness of engineered cells. Although antibiotic-based selection systems can enrich transduced populations *in vitro*, their use *in vivo* is limited by safety and regulatory constraints. To overcome these challenges, we present a drug-free selection strategy based on uridine-5′-monophosphate synthase (UMPS) auxotrophy. Efficient *UMPS* disruption via CRISPR-Cas9 rendered cells dependent on uridine supplementation for survival and concomitantly enabled the selection of knockout cells based on their acquired resistance to the chemotherapeutic prodrug 5-fluoroorotic acid. Lentiviral reconstitution of UMPS restored uridine independence, and enabled the formation of >90% gene-marked cells within 7–14 days upon uridine removal. Likewise, splitting UMPS into its two functional domains—orotate phosphoribosyltransferase (OPRT) and orotidine-5′-monophosphate decarboxylase (ODC)—for expression from separate vectors promoted the effective selection of double-transduced cells under auxotrophic conditions. Apart from robust cell enrichment, long-term stabilization of transgene-expressing cells without pharmacological intervention could be demonstrated and was superior to antibiotic selection. Our work thus establishes UMPS auxotrophy-rescue as a versatile platform for both *in vitro* and possibly *in vivo* applications, offering a safe and scalable alternative to conventional drug-based selection methods.

## Graphical abstract

**Figure:**
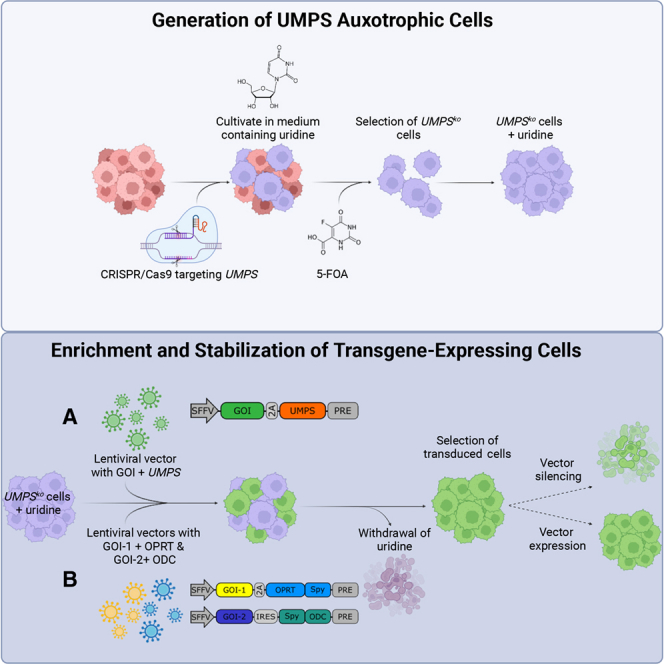

## Teaser

Maetzig and colleagues develop a lentiviral auxotrophy-rescue strategy for engineered UMPS-deficient cells that ties transgene expression to cellular fitness. This drug-free system enriches genetically modified cells to near purity and ensures stable long-term expression, offering a robust selection method for *in vitro* work and potential *in vivo* use.

## Introduction

Retroviral vectors are stable gene transfer vehicles derived from natural retroviruses and a common tool in biomedical research and cell and gene therapy.^1^ However, transgene silencing through epigenetic regulation and chromatin condensation is a natural defense mechanism that is particularly active in hematopoietic stem and progenitor cells (HSPC) and pluripotent cells and may limit therapeutic success and biological insights.^2^^,^^3^^,^^4^^,^^5^^,^^6^ A well-known example is the hematopoietic gene therapy trial of gp91Phox, in which vector expression was switched off, but the retroviral enhancer continued to fuel the uncontrolled expansion of insertional mutants.^7^^,^^8^^,^^9^ A number of strategies have therefore been developed for preventing silencing, which include modification of the primer-binding site, composite promoters and the use of naturally occurring chromatin opening and insulator elements.^10^^,^^11^^,^^12^^,^^13^^,^^14^^,^^15^ Furthermore, transgene-expressing cells might also be lost over time if the transgene itself imposes a growth disadvantage. Although these gene-marked cells can be maintained as pure cultures, even minimal contamination with non-transduced cells or the spontaneous loss of transgene expression can lead to the rapid outgrowth of transgene-negative populations. For *in vitro* experiments, the vector can be equipped with an additional antibiotic-drug-selection cassette; however, this results in more complex vector designs, lower titers, and the requirement for constant drug supply. For *in vivo* selection, the situation is even more complex as selection strategies need to impair the growth of normal cells and promote the expansion of genetically modified cells. One attempt was made with the overexpression of the O^6^-methylguanine-DNA-methyltransferase (MGMT) variant MGMT-P140K in hematopoietic stem cells (HSCs) to protect them from the toxicity of alkylating chemotherapeutic drugs.^16^^,^^17^^,^^18^^,^^19^ Despite slow enrichment of genetically modified cells, a drop in gene marking rates could not totally be prevented and possibly reflects the growth interfering effect of MGMT-P140K or deficits in HSC marking.^20^^,^^21^ Based on these limitations, a selection system for *in vitro* and *in vivo* applications appears desirable, which enables a natural growth behavior of genetically modified cells and concomitantly stabilizes vector expression without the need for repetitive or continuous administration of cytotoxic drugs.

One possibility to achieve drug-free selection is the use of genetically engineered auxotrophic cells that are defined by their inability to synthesize a specific organic compound required for their growth, necessitating its acquisition from the environment, e.g., through supplementation of the growth media. These synthetic auxotrophic cells offer the opportunity to stabilize gene marking rates by coexpression of the gene of interest with the essential gene in the absence of supplement. For example, auxotrophy for dihydrofolate reductase *(Dhfr)* is commonly used for recombinant protein production.^22^^,^^23^ We have successfully implemented this concept in our previous studies, wherein the expression of a large lentiviral fluorescent genetic barcoding (FGB) vector was stabilized through addicting cell survival to the coexpression of the *Dhfr* transgene.^24^ However, it has also become clear that the enrichment and cultivation of pure *Dhfr*^−/−^ cell populations are time-consuming and cumbersome, which calls into question its application to primary cells with limited *in vitro* cultivation potential.^24^^,^^25^^,^^26^^,^^27^

Wiebking et al. recently presented an alternative auxotrophy model based on the inactivation of uridine-5′-monophosphate synthase (UMPS) as a potential safety switch in T cell therapies.^28^ They exploited the fact that UMPS, which consists of two functional subunits, an orotate phosphoribosyltransferase (OPRT) and an orotidine-5′-monophosphate decarboxylase (ODC), catalyzes two crucial steps of the *de novo* pyrimidine biosynthesis pathway. UMPS knockout (*UMPS*^ko^) cells cannot convert orotate into uridine monophosphate (UMP), an essential uracil precursor, and thus rely on uridine supplementation for survival. Moreover, *UMPS^ko^* cells can be specifically enriched due to their inability to convert 5-fluoroorotic acid (5-FOA) into the cytotoxic drug 5-fluorouracil (5-FU).^28^ These properties would be ideal for a drug-free selection system for gene therapy and biomedical research, providing that cell survival could be addicted to the vector-mediated coexpression of UMPS.

Here, we sought to determine whether the UMPS-based safety switch could be adapted to a drug-free selection system that couples stable lentiviral transgene expression with cell survival. Our results demonstrate the efficient derivation of various human and murine *UMPS*^*ko*^ hematopoietic cell lines and primary human T cells as well as the outgrowth and stable maintenance of UMPS-vector addicted cells after cessation of uridine supplementation. Similar result can be achieved with an engineered split-UMPS design, which hinges cell survival on the simultaneous integration of two independent vectors. The UMPS auxotrophy system thus provides a user-friendly and versatile tool for the selection and long-term stabilization of transgene-expressing cells in both research and therapeutic applications.

## Results

### *Umps*^*ko*^ cells are efficiently selected by 5-FOA

**Figure 1:**
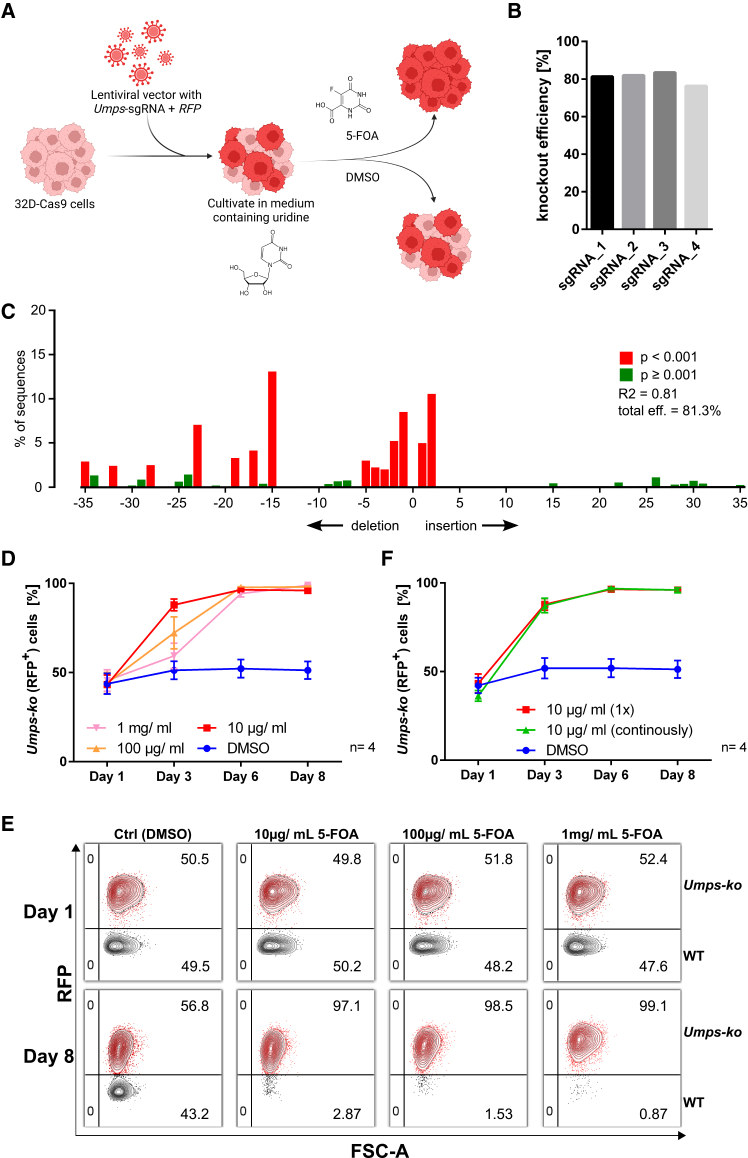
Generation of 32D *Umps*^*ko*^ cells (A) Schematic overview: 32D-Cas9 cells were transduced with a lentiviral vector encoding for red fluorescent protein (RFP) and an sgRNA targeting *Umps*, followed by 5-FOA treatment for positive selection of knockout cells. Supplementation of cells with uridine starting after transduction. (B) Representative knockout efficiencies determined by TIDE of four different *Umps*-sgRNAs. (C) Exemplary TIDE analysis of *Umps* locus after knockout with sgRNA_1. Position 0 marks the expected cleavage site with regard to the WT sequence. (D) One-time treatment of mixed 32D WT (RFP^−^) and *Umps*^*ko*^ (RFP^+^) cells with increasing 5-FOA concentrations. *n* = pooled data from 4 different sgRNAs. (E) FC plots of day 1 and day 8 of one-time treatment of mixed 32D WT (RFP^−^) and *Umps*^*ko*^ (RFP^+^) cells (sgRNA_4) with increasing 5-FOA concentrations. (F) Comparison of one-time vs. continuous treatment for selection of *Umps*^*ko*^ cells with 10 μg/mL 5-FOA. One-time 5-FOA treatment and the DMSO control identical to (D). *n* = pooled data from 4 different sgRNAs. For D and F, error bars represent mean ± SD.

To test the conditions for vector stabilization by the UMPS auxotrophy model, we first established efficient conditions for inactivating UMPS and positively selecting the resulting knockout cells. Therefore, the murine 32D myeloblast-like cell line expressing Cas9 (32D-Cas9) was transduced with lentiviral vectors encoding a red fluorescent protein (RFP) and a single guide RNA (sgRNA) targeting different locations of the *Umps* gene (Figure 1A; Figure S1). Four tested sgRNAs successfully induced small insertions or deletions (INDELs) in the *Umps* gene with a mean efficiency of ∼80% as determined by tracking of indels by decomposition (TIDE) in a representative experiment (Figures 1B and 1C).^29^ For positive selection of *Umps*^*ko*^ cells, different concentrations of 5-FOA (10 μg/mL, 100 μg/mL, and 1 mg/mL) and treatment timelines were compared in cultures supplemented with 250 μg/mL uridine from the time of transduction. Every tested 5-FOA concentration completely eradicated non-transduced WT cells (RFP^−^) within 1 week, after which pure knockout populations had been established. In contrast, no enrichment of *Umps*^*ko*^ cells (RFP^+^) was observed in the absence of 5-FOA (DMSO Ctrl) indicative of comparable proliferation rates of knockout and wildt-ype cells in the presence of uridine (Figures 1D and 1E). Importantly, increasing 5-FOA concentration impaired cell growth encouraging the use of the lowest effective concentration possible (Figure 1D). Furthermore, we compared a continuous treatment, in which the cells were cultured in media containing 5-FOA for 1 week, with a one-time treatment, in which 5-FOA was washed-out after 2 days. Both application timelines were equally efficient and a one-time treatment with 10 μg/mL 5-FOA was already sufficient to completely eradicate all wild-type cells (Figure 1F).

### The UMPS-knockout can be efficiently introduced by CRISPR-Cas9 RNPs

**Figure 2:**
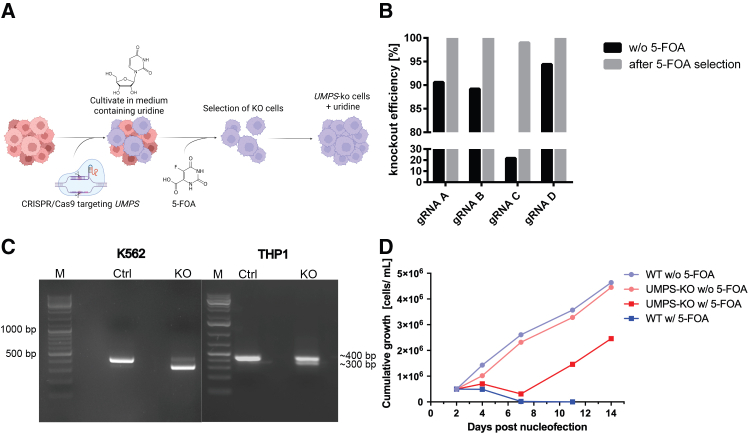
*UMPS*^*ko*^ by CRISPR-Cas9 in murine and human leukemia cell lines (A) Schematic overview: nucleofection of Cas9/gRNA RNPs to induce knockout of *Umps/UMPS*, followed by 5-FOA treatment for positive selection of knockout cells. Supplementation of cells with uridine starting after nucleofection. (B) Knockout efficiencies of four different gRNAs targeting *Umps* in H9M cells before and after 5-FOA selection (1 × 10 μg/mL) as determined by TIDE in a representative experiment. (C) Gel pictures of PCR amplicons of the *UMPS* locus in WT (Ctrl) and *UMPS*^*ko*^ (KO) K562 and THP1 cells. *UMPS*^*ko*^ cells were generated using two gRNAs. (D) Representative growth curve (based on trypan blue cell counts) of THP1 WT and *UMPS*^*ko*^ cells with or without one-time 5-FOA treatment (10 μg/mL).

Having confirmed the feasibility of *Umps* knockout in 32D-Cas9 cells and the efficient positive selection of knockout cells by 5-FOA, we next developed an *Umps* knockout approach using nucleofection of Cas9-gRNA ribonucleoparticles (RNPs) (Figure 2A). This method can be applied to a variety of cell types and has the advantage of being transient, thus reducing the risk of long-term off-target effects. Four tested gRNAs targeting different regions of the *Umps* gene (Figure S1) successfully induced INDELS in primary *Hoxa9* and *Meis1* (H9M) transformed murine acute myeloid leukemia (AML) cells,^30^ which allow for long-term *in vitro* cultivation and had previously been used by our group to validate the *Dhfr*-auxotrophy approach. In these cells, *Umps* INDEL frequencies ranged from 20% to 95%. Subsequent one-time 5-FOA treatment increased INDEL frequencies to 98%–100%, even for gRNA “C” that initially resulted in the lowest knockout rate (Figure 2B).

Next, we explored the possibility of generating human *UMPS*-knockout cells with high efficiency by combining two gRNAs for nucleofection as exemplified for K562 and THP1 leukemia cell lines (Figure S1).^31^ PCR of the target locus followed by sequencing confirmed the successful deletion of the ∼100 bp region in between the two gRNA recognition sites (Figure 2C, ∼300 bp band). In addition, sequencing of the 400 bp parental target locus showed a high INDEL frequency induced by either gRNA alone, represented by a complete loss of wild-type sequence in TIDE. Prior to 5-FOA selection, THP1 WT cells and cells nucleofected for *UMPS* knockout exhibit comparable proliferation rates. As already observed in 32D cells (Figure 1D), medium supplementation with 250 μg/mL uridine completely compensated for the loss of UMPS activity (Figure 2D). Upon one-time treatment with 10 μg/mL 5-FOA, cell proliferation stagnated and finally wild-type cultures were eradicated 5 days after treatment. In contrast, positively selected THP1 *UMPS*^*ko*^ cells recovered and their proliferation behavior returned to that of untreated cells (Figure 2D). The eradication of wild-type cells by 5-FOA treatment was confirmed by the loss of wild-type signal in sequencing. In our data, 5-FOA selection had no significant preference for cells containing either the deletion produced by two gRNAs or an INDEL produced by only one gRNA (data not shown).

Taken together, we successfully demonstrated the feasibility of generating murine and human *Umps/UMPS*-knockout lines, to stably maintain them in culture through supplementation with uridine and trigger their rapid positive selection through 5-FOA treatment.

### Overexpression of UMPS rescues auxotrophy and promotes cell expansion in the absence of uridine

**Figure 3:**
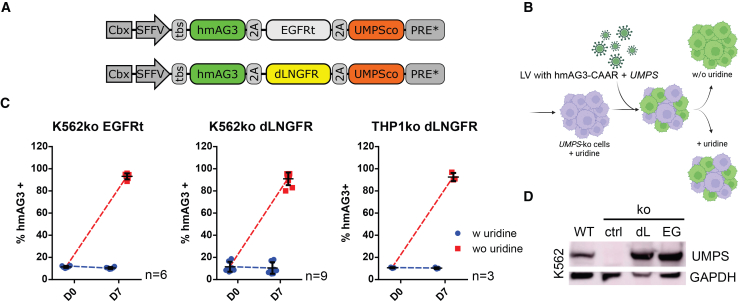
Overexpression of *UMPS* rescues auxotrophy (A) Lentiviral vectors consisting of a silencing resistant spleen-focus forming virus promoter (SFFV) and a polycistronic expression cassette linking the green fluorescent humanized monomeric Azami green (hmAG3) fluorescent protein, a truncated surface marker (EGFRt or dLNGFR) as part of a chimeric antigen array (CAAR) and h*UMPS*co by 2A peptides. (B) Schematic overview: *UMPS*^*ko*^ cells are first transduced with a lentiviral vector encoding for a hmAG3-CAAR-UMPSco cassette (A) followed by cultivation with or without uridine. (C) UMPS-transduced K562 and THP1 *UMPS*^*ko*^ cells (hmAG3^+^) are enriched from mixed cultures by uridine withdrawal. Gene marking levels were first measured on day 0 (D0), and enrichment of cells through uridine withdrawal was evaluated on day 7 (D7) using FC. Each experiment was performed in three biological replicates. Experiments using K562 *UMPS*^ko^ cells expressing EGFRt were conducted twice, those with dLNGFR were performed three times, and experiments with THP1^ko^ cells were carried out once. (D) Western blot analysis of K562 wildtype (WT) cells, K562 *UMPS*^*ko*^ cells (ctrl), and K562 *UMPS*^*ko*^ cells transduced with the UMPSco vector coexpressing either dLNGFR (dL) or EGFRt (EG). Individual data points are shown; error bars represent mean ± SD.

Next, we wondered, if the lentiviral expression of *UMPS* would rescue *UMPS*^*ko*^ cells and lead to positive selection of transduced cells upon withdrawal of uridine supplementation. To investigate this, K562 *UMPS*^ko^ cells, previously selected by 5-FOA treatment, were transduced with a lentiviral vector encoding for a polycistronic expression cassette consisting of the humanized Azami green fluorescent protein (hmAG3), a surface marker (truncated EGFRt or dLNGFR), and human UMPS (Figure 3A). Notably, we employed a human codon-optimized *UMPSco* cDNA for this purpose to achieve maximal protein expression from a compound promoter consisting of the Cbx3 anti-silencing element and the strong spleen-focus forming virus (SFFV) promoter (Figure 3A; Figure S2). After an initial assessment of gene marking rates, cells were subsequently split and cultivated in the presence and absence of uridine, respectively, for another 7 days (Figure 3B). Flow cytometric analyses demonstrated the almost complete enrichment of transduced cells from an initial 11.45% ± 0.93 to 93.15% ± 2.7 (EGFRt) and 11.51% ± 4.5–90.94% ± 5.73 (dLNGFR), respectively. In contrast, the gene marking rate was stable (10.47% ± 0.87 for EGFRt and 10.44% ± 5.19 for dLNGFR) in cultures grown in uridine-containing media, confirming the selective pressure exerted by uridine withdrawal (Figure 3C). Similar results were obtained in THP1 *UMPS*^ko^ cells after transduction with the dLNGFR-UMPSco vector (Figures 3A–3C). Western blot analysis verified the loss of UMPS signal in K562 *UMPS*^ko^ cells as well as the restoration of UMPS expression upon transduction (Figure 3D).

In summary, lentiviral *UMPS* overexpression in *UMPS*^ko^ cells rescues auxotrophy and excerts a rapid growth advantage in the absence of uridine.

### Enrichment of FGB-UMPS color-coded *UMPS*^*ko*^ cells

**Figure 4:**
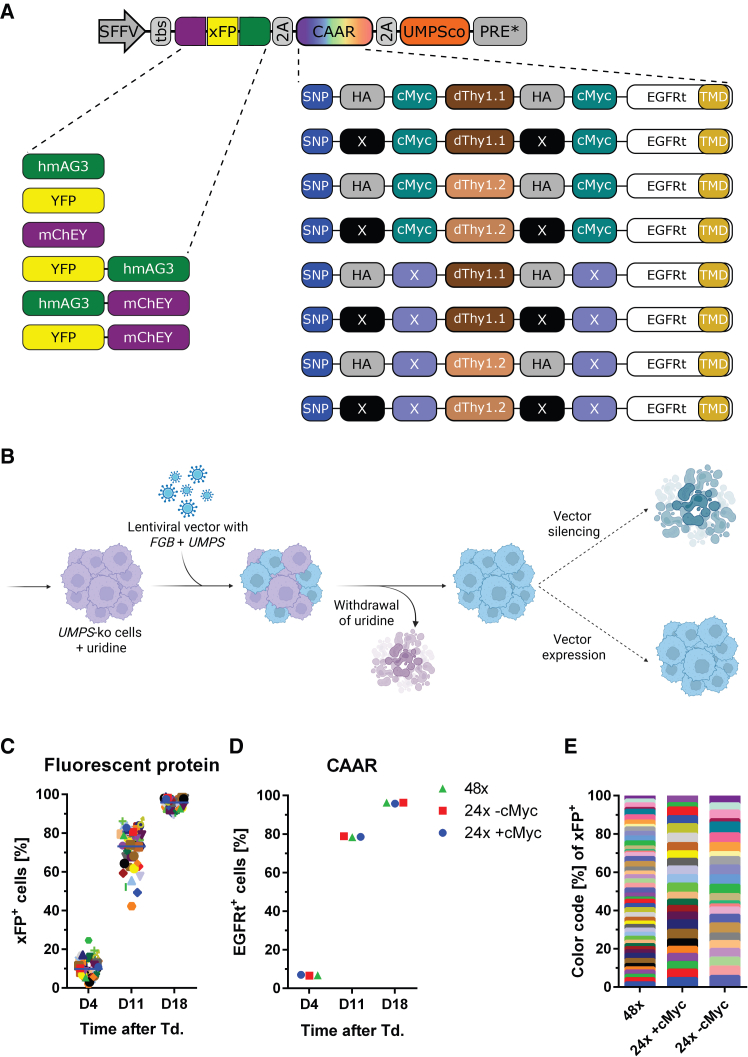
Enrichment of FGB-UMPS color-coded *UMPS*^*ko*^ cells (A) SFFV-driven lentiviral vector for coexpression of fluorescent genetic barcode (FGB) and UMPS transgene (FGB-U). The FGB vector consists of either one or two fluorescent proteins (xFP: hmAG3, YFP, mChEY) and the chimeric antigen array (CAAR) generating a total of 48 color codes. The CAAR includes a truncated epidermal growth factor receptor (EGFRt) with its transmembrane domain (TMD) fused to permutations of antibody binding (dThy1.1, HA, and cMyc) and non-antibody binding (dThy1.2 and X) versions of a truncated CD90 epitope, hemagglutinin (HA) and cMyc tags. (B) Schematic overview: *UMPS*^*ko*^ cells are first transduced with a lentiviral vector encoding for an FGB-UMPS cassette (A) followed by uridine withdrawal to enrich vector-expressing cells. Vector silencing would result in cell death. (C) THP1 *UMPS*^*ko*^ cells were individually transduced with each one of the 48x FGB-U vectors. Four days after transduction, uridine was washed out and enrichment of transduced cells was analyzed by FC using xFP expression. The mean of all individual data points is indicated. (D) Individual FGB-U THP1 *UMPS*^*ko*^ populations were pooled to samples containing 24 or 48 color codes. Enrichment of transduced cells upon uridine withdrawal was analyzed by FC using CAAR expression (EGFRt^+^, individual data points for pooled sample analysis). (E) Color code distribution in mixed samples consisting of all 48 or 24 (+/− cMyc) color codes.

Fluorescence-based tracking approaches such as lentiviral FGB enable the flow cytometric longitudinal analysis of color-coded populations or cell clones in mixed samples.^24^^,^^32^^,^^33^ This allows the simultaneous analysis of numerous transduced cells, whereby the quality and complexity of the data depend heavily on the stability of vector expression and the number of available color codes. Since we observed an EGFRt-mediated growth disadvantage of FGB-transduced cells in previous color-coding experiments,^24^ we next cloned a series of 48 FGB vectors with unique color codes (48x FGB), where DHFR was replaced by a UMPS coexpression cassette (48x FGB-U) (Figure 4A). This allowed to monitor the enrichment of color-coded cells in the absence of uridine, the stability of vector expression, and the effectiveness of long-term vector addiction on a large number of samples in parallel (Figure 4B). At the same time, these vectors are used to determine whether the integrity of complex vector cassettes can be maintained via UMPS addiction.

Thus, THP1 *UMPS*^ko^ cells were individually color-coded using the FGB-U vectors with an initial transduction rate of ∼10%. Uridine deprivation positively selected transduced cells and resulted in pure *FGB*-*UMPS*-expressing cultures after 2 weeks (Figures 4C and 4D). Importantly, flow cytometric deconvolution enabled the unequivocal identification of all 48 input color codes as well as sublibraries consisting of 24 color codes from mixed samples (Figure 4E; Figure S3). These data demonstrate the applicability of the UMPS system for stabilizing transgene expression in complex vector cassettes and a comparable selection efficiency of a large number of constructs.

### ODC/OPRT split vector system rescues UMPS auxotrophy

**Figure 5:**
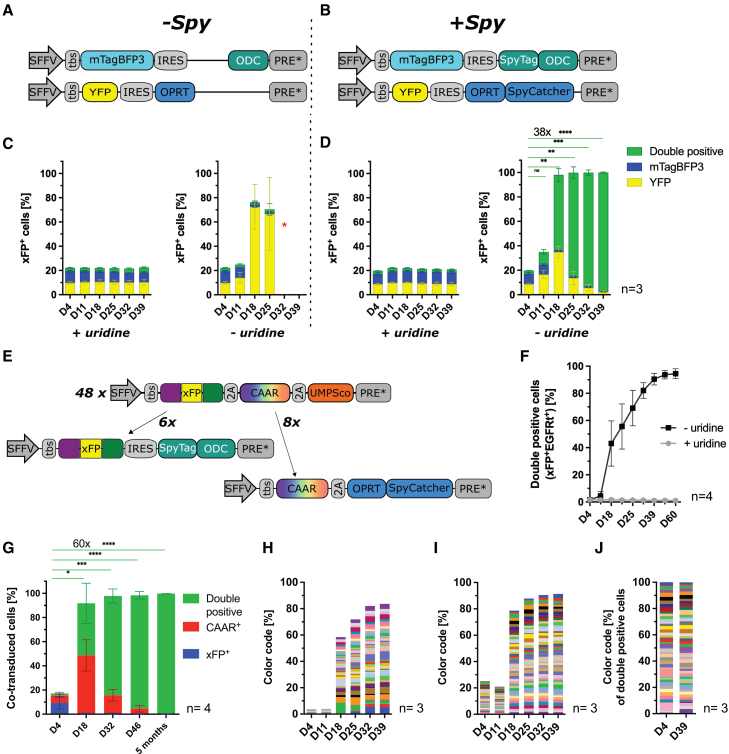
ODC/OPRT split vector system rescues UMPS auxotrophy and allows for complex color coding with reduced vector numbers (A and B) Lentiviral vectors for coexpression of ODC and mTagBFP3 or OPRT and YFP (A) without or (B) with addition of SpyTag and SpyCatcher (+/−Spy) domains, respectively. (C and D) THP1 *UMPS*^*ko*^ cells were co-transduced with the ODC and OPRT vectors. Transduced cells were cultured either in the presence or absence of uridine. Analysis of population sizes of the four resulting groups (non-transduced, only YFP^+^, only mTagBFP3^+^ or double transduced cells) was performed by FC at the indicated days after transduction. ∗-Spy cultures without uridine did not survive beyond D25. Significant changes of the double-positive cell populations between day 4 and later time points are indicated by asterisks (∗*p* ≤ 0.05, ∗∗*p* ≤ 0.01, ∗∗∗*p* ≤ 0.001, ∗∗∗∗*p* ≤ 0.0001, ns: *p* > 0.05, two-way ANOVA). (E) The FGB-U lentiviral vector design for coexpression of fluorescent proteins (xFP), a cell surface marker (CAAR) and UMPS requires 48 vectors to generate 48 color codes. Using the split vector design, all 48 color codes can be generated by co-transduction of two vectors, only requiring a total of 14 vectors: 6 vectors encoding a xFP-IRES-SpyTag-ODC cassette and 8 vectors encoding a CAAR-2A-OPRT-SpyCatcher cassette. (F–J) THP1 *UMPS*^*ko*^ cells were transduced in separate wells with 48x vector combinations, each consisting of an xFP and a CAAR expressing construct. Uridine supplementation was discontinued four days after transduction. Longitudinal flow cytometric analysis determined xFP and CAAR (EGFRt) expression. Color codes were identified in pooled samples through detection of CAAR (EGFRt, cMyc, HA, and Thy1.1) and xFP (hmAG3, YFP, and mChEY) markers. (F) Enrichment of double transduced cells (xFP^+^& EGFRt^+^) in the presence and absence of uridine. *n* = 4 different vector combinations. (G) Co-transduction of the ODC/OPRT vectors resulted in mix of single positive (xFP^+^ or CAAR^+^), double-positive or untransduced cells before uridine withdrawal. 60-fold enrichment of double transduced cells and stable expression of both vectors 5 months after transduction. *n* = 4 different vector combinations. Significant changes of the double-positive cell populations between day 4 and later time points are indicated by asterisks (∗*p* ≤ 0.05, ∗∗*p* ≤ 0.01, ∗∗∗*p* ≤ 0.001, ∗∗∗∗*p* ≤ 0.0001, two-way ANOVA). (H and I) Color code distribution in pooled samples with (H) low compared to (I) high initial transduction rate, cultivated without uridine from day 4 after transduction. Pools were stained and analyzed by FC once a week for their color code expression. Mean of *n* = 3 pools of 48x ODC/OPRT-transduced samples. (J) Relative color code distribution, defined as percentage of double-positive (xFP^+^CAAR^+^) cells, in pooled samples on day 4 compared to day 39 after transduction (compare to I). Uridine supplementation was ended on day 4. Mean of *n* = 3 pools of 48x ODC/OPRT-transduced samples. Error bars indicate mean ± SD.

The use of complex transgene cassettes, such as FGB vectors, is complicated by their size-dependent negative influence on the titer and the overall packaging limit of lentiviral vectors.^34^^,^^35^ Although splitting the transgene cassette across multiple vectors should lead to an increase in titer, the expected low frequency of cells with multiple vector integrations limits the use of cotransduction approaches.^36^ Similar limitations also exist in biomedical research when investigating different transgene combinations or aiming for biallelic recombination events. Given the independent expression of both UMPS subunits in bacteria,^37^^,^^38^ we explored the opportunity to select for double-transduced cells with vectors encoding for *ODC* (linked to mTagBFP3) and *OPRT* (linked to YFP).^37^^,^^38^^,^^39^ Due to the reduced stability of the monomeric subunits in human cells,^40^ we furthermore investigated the advantage of incorporating dimerization domains by employing the SpyTag/SpyCatcher system (SpyTag003 and SpyCatcher003 [+Spy]) (Figures 5A and 5B; Figure S4).^41^^,^^42^ This showed that only “+Spy”-transduced cells exhibited a significant enrichment and expressed both vectors in >95% of the cells upon uridine withdrawal. This corresponds to a 38-fold enrichment, clearly supporting the hypothesis that fused UMPS subunits are necessary for efficient selection (Figures 5C and 5D). In contrast, “–Spy”-transduced cells cultured in the absence of uridine did not survive beyond 25 days, further underscoring the requirement for fused UMPS subunits to confer uridine independence. Since enrichment was slower compared to UMPS-transduced cells, we investigated if withdrawal of uridine already at the time of transduction would be feasible and could increase enrichment speed. Performing transduction in the absence of uridine led to an enrichment of double-transduced cells as early as day 4, compared to cells supplemented with uridine. Furthermore, the proportion of double-positive cells increased by over 90% at day 18, compared to day 25, when uridine was withdrawn on days 0 or 4, respectively (Figure S5A).

### A 14x split FGB-U vector series enables the selection of 48 color codes

We next investigated the feasibility of splitting larger vector cassettes into functional units as exemplified for the 6 fluorescent marker cassettes (coupled to SpyTag-ODC) and 8 surface marker cassettes (*aka* CAAR, chimeric antigen array) (coupled to OPRT-SpyCatcher) of the FGB vector series (Figure 5E). These 14 vectors still allow the creation of 48 color codes by combinatorial transduction that would typically lead to a very low frequency of xFP^+^CAAR^+^ cells.^33^^,^^36^ Furthermore, this strategy reduced the size of the individual vectors (UMPS vector: ∼11.5–12.2 kb vs. ODC vector: ∼9.5 kb and OPRT vector: ∼10.2 kb) while increasing viral titers (Figure S5B). We subsequently produced these 14 supernatants and transduced THP1 *UMPS*^ko^ cells with all possible ODC/OPRT vector combinations in 48 separate wells. As hypothesized, uridine withdrawal in separate cultures led to the enrichment of double-transduced cells from ∼2% to ∼80% within 3 weeks (Figures 5F and S5C). In these cultures, also OPRT-transduced (CAAR^+^) single-positive cells expanded but were subsequently replaced by xFP^+^CAAR^+^ cells underlining the requirement for full UMPS activity for sustained proliferation (Figures 5F and 5G). A similar dynamic could also be observed in mixed cultures from the 48 individually color-coded samples (Figure 5H). Using a 10-fold higher initial transduction rate (∼22% double transduced) allowed the reliable identification of all 48 color codes (∼0.5% of cells/color code) already 4 days after transduction and the relative color code distribution remained stable even during expansion in the absence of uridine (Figures 5I and 5J). Together, this demonstrates that the split-UMPS vector addiction approach provides greater vector design flexibilities than the single-vector approach and benefits from comparable selection efficiencies—albeit with slower kinetics—and survival properties of transduced *UMPS*^*ko*^ cells.

### UMPS-mediated selection outperforms antibiotic selection strategies

**Figure 6:**
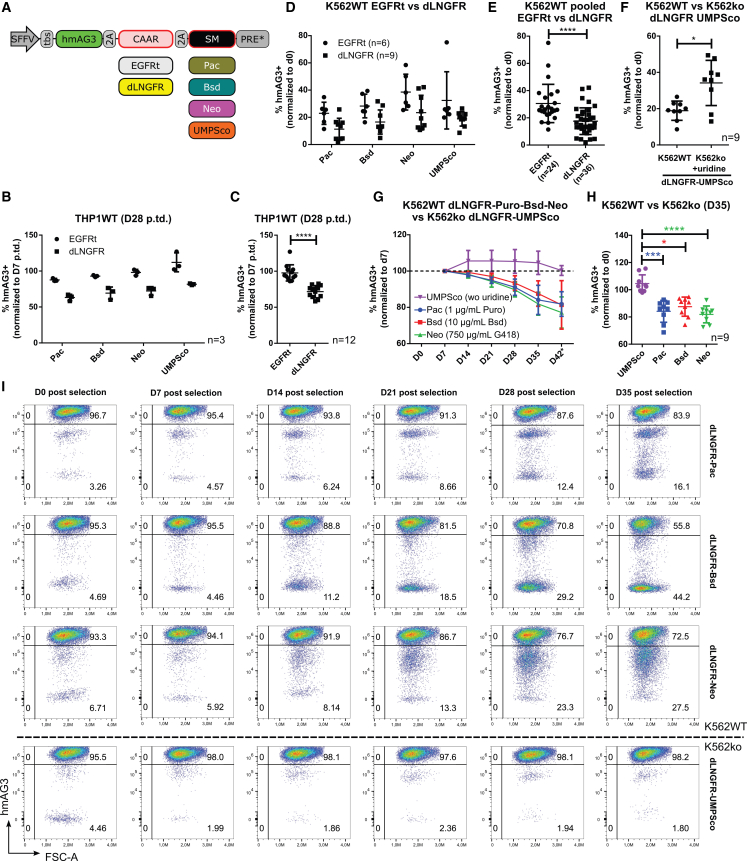
UMPS outperforms conventional antibiotic selection (A) Lentiviral vector encoding for the green fluorescent protein hmAG3 and a chimeric antigen array (CAAR) containing either the EGFRt or dLNGFR surface receptor followed by a selection marker (SM). The selection marker was made up by puromycin N-acetyltransferase (Pac), blasticidin S deaminase (Bsd), amino 3′-glycosyl phosphotransferase (Neo) and UMPSco, respectively. (B) Assessment of vector stability in THP1 wildtype (WT) cells. Normalized gene marking rates between EGFRt and dLNGFR vector transduced cells were compared 28 days post transduction (p. td.). A single experiment was performed in triplicates (*n* = 3). (C) Pooled data from B demonstrate a significantly faster loss of dLNGFR-expressing THP1 cells (Mann-Whitney U test). (D) Comparison of transgene-maintenance in K562 WT cells after transduction with EGFRt and dLNGFR vectors for coexpression of Pac, Bsd, Neo, and UMPSco, respectively. Data from two (EGFRt) and three (dLNGFR) independent experiments performed in triplicate are shown. (E) Pooled data from (D). demonstrate a significantly faster loss of dLNGFR-expressing K562 WT cells (MannWhitney U test). (F) Comparison of transgene maintenance between dLNGFR-UMPSco vector-transduced K562 WT cells and K562 *UMPS*-knockout (ko) cells supplemented with uridine. Data from three independent experiments performed in triplicate are shown. Significance was determined using a Mann-Whitney U test. (G) Longitudinal comparison of gene-marking rates between dLNGFR vector-transduced (Pac, Bsd, and Neo) K562 WT cells and dLNGFR-UMPSco vector–transduced K562 *UMPS*^*ko*^ cells. Day 0 (D0) corresponds to the first flow-cytometric analysis assessing gene-marking rates before the start of selection. Selection was performed between day 0 and day 7 (D7). Day 7 gene-marking rates were used for normalization and were arbitrarily set to 100%. For each construct, nine data points (day 0 to day 35) and six data points (day 42∗) were obtained from three and two independent experiments, respectively. (H) Comparison of transgene maintenance between dLNGFR-UMPSco vector-transduced *UMPS*^*ko*^ cells and dLNGFR vector-transduced K562WT cells (Pac, Bsd, and Neo) 28 days after selection (corresponding to day 35 of tracking from (G)). Significance was determined using a Kruskal-Wallis test followed by Dunn’s multiple-comparison test. (I) Representative plots of the longitudinal flow-cytometric analysis of transduced K562 and K562ko cells. Day 0 represents the first time point after completion of the selection process, corresponding to day 7 (D7) in (G). Error bars indicate mean ± SD.

Despite the effectiveness of UMPS-mediated selection, it remains necessary to determine whether the additional effort required to generate UMPS-deficient cells is justified, or whether conventional drug-resistance selection methods provide a more practical approach. For this reason, we cloned polycistronic vectors equipped with either hmAG3 and EGFRt or dLNGFR CAARs in combination with either *UMPS*co or cDNAs conveying resistance against puromycin (Pac, puromycin N- acetyltransferase), blasticidin (Bsd, blasticidin S deaminase), and neomycin (Neo, neomycin phosphotransferase II) respectively (Figure 6A). Transduction of these constructs into THP1 cells resulted in a more rapid loss of gene marking for dLNGFR-expressing cells than for EGFRt-expressing cells over time (Figure 6B). This difference became significant after pooling all EGFRt and dLNGFR samples (Figure 6C). Similar results were observed in K562 cells (Figures 6D and 6E), although the degree of transgene loss was greater, presumably due to the higher proliferation rate of K562 cells compared with THP1 cells. The relatively rapid loss of the dLNGFR vectors in K562 cells suggests that this cell line is a suitable system for comparing different selection strategies aimed at maintaining sustained vector expression. An initial comparison between K562 WT cells transduced with dLNGFR-UMPSco vectors and uridine-supplemented K562 *UMPS*^*ko*^ cells suggested slightly improved vector maintenance in the auxotrophic cells (Figure 6F).

Subsequently, K562 cells transduced with the dLNGFR surface marker for coexpression with Pac, Bsd, and Neo were subjected to flow cytometric analysis before and after antibiotic selection for 7 days (Figure S6). Longitudinal tracking of gene marking rates based on hmAG3 expression in these cultures revealed a continuous decline. In contrast, K562 *UMPS*^*ko*^ cultures transduced with the dLNGFR-UMPSco vector maintained a uniformly high gene-marking rate (Figure 6G), which became significantly higher compared to all drug-selected cultures as early as 35 days of tracking (corresponding to day 28 after antibiotic wash-out) (Figure 6H). Closer inspection of the flow cytometry data showed consistently high hmAG3 frequencies in K562 *UMPS*^*ko*^ cells, whereas drug-selected samples progressively developed weakly fluorescent and fluorescence-negative cell populations (Figure 6I).

Collectively, these findings highlight the superior effectiveness of UMPS-mediated selection in preserving both the frequency and intensity of transgene expression, outperforming conventional drug-based selection approaches.

### UMPS overexpression rescues the growth defect in auxotropic T cells

**Figure 7:**
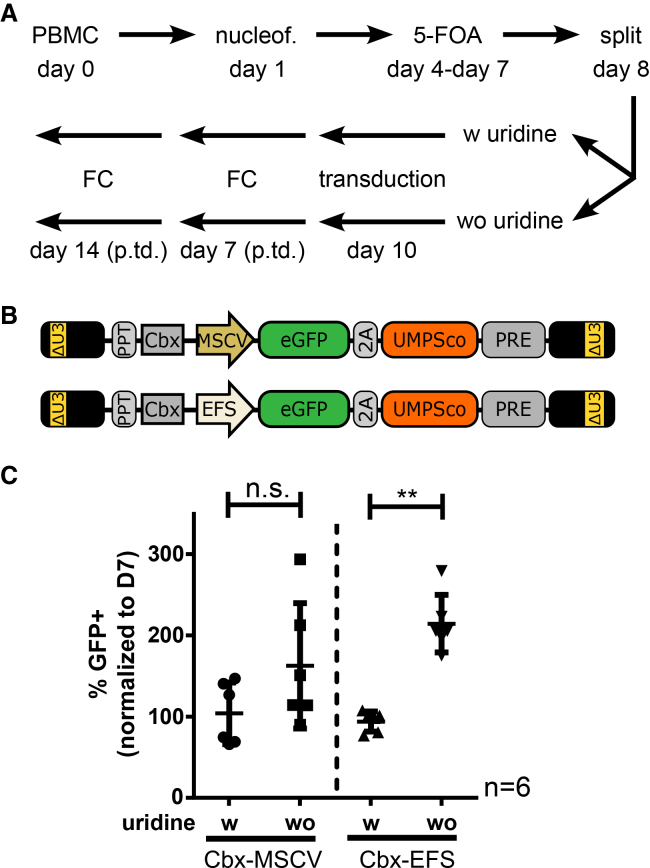
UMPS enriches primary T cells (A) Experimental outline for the generation and rescue of auxotrophic primary human T cells. (B) Lentiviral vectors for the coexpression of eGFP and *UMPS*co from compound promoters consisting of the Cbx-derived anti-silencing element and either a murine stem cell virus (MSCV) or a short version of the human eukaryotic elongation factor 1 alpha (EFS) promoter. (C) Gene marking rates of T cell cultures were analyzed by flow cytomertry (FC) 14 days after transduction (td.) with either Cbx-MSCV or Cbx-EFS vectors in the presence or absence of uridine. Data show the normalized gene marking rate to the day 7 analysis for two independent experiments performed in triplicate. Significance was determined by Mann-Whitney U test. Error bars indicate mean ± SD.

Finally, we investigated whether the UMPS rescue strategy could also be applied to primary cells. This represents a greater challenge, as culture duration is inherently limited and proliferation is often slow. To this end, human peripheral blood mononuclear cells (PBMCs) were placed under culture conditions supporting T cell survival, and nucleofection was performed using guides UMPS-7 and UMPS-1.AA (Figure S1; Table S1) the following day before enriching *UMPS*^*ko*^ cells using two cycles of 5-FOA selection. Subsequently, cultures were propagated either in the presence or absence of uridine prior to lentiviral transduction (Figure 7A). For this purpose, constructs coexpressing eGFP and UMPSco under the control of Cbx-MSCV or Cbx-EFS promoters were used (Figure 7B). Early uridine withdrawal was intended to accelerate the selection process (Figure S5A). Although we observed a trend toward enrichment of Cbx-MSCV-modified cells between days 7 and 14 after transduction, this did not reach significance when compared with cultures maintained in the presence of uridine (Figure 7C). However, analysis of Cbx-EFS-transduced cultures revealed a significantly greater expansion in the absence of uridine (Figure 7C), demonstrating that the UMPS-auxotrophy rescue strategy is functional in primary cells.

## Discussion

In this work, we leveraged the power of a combined auxotrophy—lentiviral rescue approach to establish a universally applicable drug-free selection system for both research and therapy. The basis for this is the *UMPS* knockout, originally described as a “safety switch”^28^ that could be exploited for the enrichment of gene-modified cells, stabilization of vector expression, and suppression of variegation.^3^^,^^24^ In our previous research on a *Dhfr*-based auxotrophy and vector addiction approach, two clear limitations emerged. Firstly, the provision of essential supplements should allow the knockout cells to grow at a normal rate and secondly, enrichment of knockout cells should be easily possible to accelerate their use in functional assays.^24^ From this point of view, the Porteus group had already demonstrated the *in vitro* and *in vivo* maintenance of *UMPS*^*ko*^ T cells, when supplemented with uridine, as well as their enrichment through 5-FOA treatment.^28^ Considering that, we expanded the same strategy to the generation of murine and human leukemia knockout cell lines (Figures 1 and 2); our findings indicate that CRISPR-Cas9-mediated *UMPS* knockout followed by 5-FOA counter-selection may be a broadly applicable strategy for genome engineering in diverse mammalian cells. However, it should be considered that the efficiency of *UMPS* disruption, the proliferative state of the target cells, and their sensitivity to fluoropyrimidines are expected to critically influence performance. Slowly dividing or terminally differentiated cells may show reduced susceptibility to 5-FOA because of low UMPS expression and a greater reliance on uridine salvage pathways.^43^^,^^44^ Likewise, tumor cell lines with pre-existing 5-FU resistance may display impaired 5-FOA-mediated negative selection, as 5-FOA and 5-FU converge on the same fluoropyrimidine metabolites and share common resistance mechanisms.^45^ Finally, heterogeneous primary samples may require prior enrichment of defined subsets, such as HSC,^46^^,^^47^ to ensure a uniform response to 5-FOA. These considerations should guide the implementation of UMPS-based counter-selection across different experimental systems.

Apart, it was of utmost importance to demonstrate auxotrophy rescue though vector-mediated UMPS expression. Strikingly, we created multiple coexpression vectors with h*UMPS*co typically in the 3′ position, and observed a rapid increase in the percentage of SFFV-driven vector-expressing cells upon uridine withdrawal, and the production of >90% gene-marked cultures within 7–14 days (Figures 3 and 4). Importantly, this compares to the selection time span required for commonly employed antibiotics (Figure S6).^48^ The UMPS auxotrophy/overexpression approach thus offers the opportunity to trigger cell expansion in gene transfer scenarios in which only a small number of gene-marked cells are produced due to vector dose/copy number limitations or difficult-to-transduce target cells. In such cases, simple removal of uridine may suffice to generate therapeutically relevant frequencies of genetically corrected cells.

Before UMPS vectors can be used more widely, typical biosafety benchmarks must be met. In this regard, we have already verified that h*UMPS*co-transduced cells do not gain a growth advantage in the presence of uridine (Figures 2D, 3C, 5C, 5D, and 5F). This suggests that the increase in UMPS-transduced cells following uridine withdrawal is mainly driven by the expected loss of non-transduced cells. Nevertheless, we observed a slightly slower decline in vector expressing K562 *UMPS*^ko^ cells cultured with uridine compared to K562 WT cells (Figure 6F), so that genotoxicity studies will be required prior to any gene therapeutic application,^49^^,^^50^^,^^51^ which were clearly beyond the scope of this work.

One of the main challenges in applying the UMPS auxotrophy/rescue system to hematopoietic gene and cell therapies lies in the time required to generate pure *in vitro*-knockout cultures and their subsequent transduction. Although we routinely follow a protocol spanning approximately 20 days—including 4 days from nucleofection to the initiation of 5-FOA treatment, 6 days for selection, and an additional 7–14 days for enrichment of transduced cells—we believe this timeline can be substantially reduced. A promising strategy involves initiating selection earlier and shortening its duration by increasing the 5-FOA concentration, as previously demonstrated for puromycin-based selection in CD34^+^ cells.^52^ The objective would be to complete transduction within nine days, followed by a culture period of up to three additional days. This would align the *ex vivo* culture duration with established HSC expansion protocols.^53^^,^^54^ However, such an approach would require *in vivo* selection of gene-modified cells with the potential to regulate timing and dynamics through the administration or withdrawal of uridine.^28^ We have taken an initial step in this direction by establishing *in vitro* selection of human T cells (Figure 7). Although this proof-of-principle demonstrates the feasibility of the auxotrophy-rescue approach in primary cells, further optimization of the protocol will be required to accelerate the selection process and increase overall cell yield.

As a further innovation, we developed a split-vector system that divides *UMPS* into its functional subunits and reconstitutes the full-length enzyme in target cells through covalent linkage of OPRT and ODC (Figure 5). This modular design enables the separation of large transgene cassettes into two smaller vectors, facilitating combinatorial applications such as the generation of color-coded cells using paired FGB vectors encoding fluorescent and surface marker cassettes (Figures 5E–5J). This approach is conceptually related to recently introduced split selectable markers, in which protein *trans*-splicing aides to the reconstitution of full-length antibiotic resistance genes for the selection of specific phenotypes, such as biallelic recombinants.^55^ Similar experiments could possibly also be carried out with split-UMPS constructs, but would allow for the drug-free selection of double-edited cell populations. It should be noted that the selection of pure OPRT^+^ODC^+^ cell populations is currently still slower compared to cells transduced with a single UMPSco vector (Figures 3C and 5F; Figures S5A and S6G). Since this is due in particular to the emergence and subsequent gradual loss of OPRT^+^ODC^−^-expressing cells, vectors that provide a lower expression level might already suffice to accelerate the clearance/prevent the formation of these cells. Regardless, UMPS-based selection supports more sustained and higher transgene expression than conventional pharmacological methods (Figures 6G–6I), making this approach particularly suitable for experiments that require stable and comparable expression levels. The minimal effort needed to establish the *UMPS* knockout and the corresponding selection conditions therefore appears well justified. Taken together, this work extends the use of UMPS auxotrophy as a safety switch to its application as a potent drug-free selectable marker for gene and cell therapy.

## Material and methods

### Cultivation of cells

32D-Cas9, K562, and THP1 were cultured in RPMI 1640 (PAN-Biotech, Aidenbach, Germany) supplemented with 10% heat-inactivated fetal bovine serum (FBS; Sera Plus or FBS Standard, PAN-Biotech) and 1% penicillin/streptomycin (P/S) (Gibco/ThermoFisher Scientific, Carlsbad, USA). 32D-Cas9 media was supplemented with 5 ng/mL mIL-3 (Peprotech, Hamburg, Germany).

Murine transformed bone marrow cells overexpressing *Hoxa9* and *Meis1* (H9M) were generated as previously published and cultivated in Dulbecco’s modified Eagle’s medium (DMEM) (Gibco) supplemented with 15% FBS (Sera Plus or FBS Standard, PAN-Biotech), 1% P/S (Gibco), 1% sodium pyruvate (PAN-Biotech), 20 ng/mL murine stem cell factor (mSCF), 10 ng/mL human IL-6, and 6 ng/mL murine IL-3 (all cytokines from Peprotech).^24^

Human PBMCs were obtained with informed consent and used in accordance with approval from the local ethics committee. PBMCs were cultivated in T cell-inducing culture conditions consisting of TexMACS medium (Miltenyi Biotech) supplemented with 3% human AB serum (c.c.pro GmbH, Oberdorla, Germany), 1% P/S, 12.5 ng/mL hIL-15, and 12.5 ng/mL hIL-7 (both from Peprotech). The day after nucleofection, T cell proliferation was induced by addition of anti-hCD3 (50 ng/mL) and anti-hCD28 (500 ng/mL) antibodies (both BioLegend) for 24 h.

All cells were incubated in humidified incubators at 37°C and 5% CO_2_. 250 μg/mL uridine (Merck, Darmstadt, Germany) was added to UMPS-knockout cells. For the enrichment of UMPS-transduced cells, supplementation of the media with uridine was stopped 4–7 days after transduction if not indicated otherwise.

For assessment of proliferation, live cells were counted in a counting chamber using trypan blue (Gibco). Cells were re-seeded if necessary.

### Lentiviral vector cloning

sgRNAs (Table S1) were cloned into SGL40C.EFS.RFP657.pre (kindly provided by Dirk Heckl; Addgene plasmid # 69147).^56^ A codon-optimized human *UMPS* cDNA sequence (h*UMPS*co) was ordered as GeneArt String DNA fragment (Thermo Fisher Scientific, Carlsbad, USA), subcloned and sequence verified. For the generation of the 48x FGB-(U) vector series (pRRL.PPT.nXC.2xBbsI.Cbx-SF.tbs.xFP-P2A-CAAR-EGFRt-E2A-hUMPSco.pre∗.SIN-BCx) the *DHFR* transgene in the 48x FGB-(D) vectors (pRRL.PPT.nXC.2xBbsI.Cbx-SF.tbs.xFP-P2A-CAAR-EGFRt-E2A-hDHFRco.pre∗.SIN-BC^24^) was replaced by h*UMPS*co. Likewise, h*UMPS*co was cloned into the pRRL.PPT.Cbx3del.EFS.eGFP.PRE (Addgene plasmid # 110834) backbone to generate pRRL.PPT.Cbx3del.EFS.eGFP-T2A-hUMPSco.PRE.

Spy constructs were cloned by first PCR amplifying the *ODC* and *OPRT* domains from h*UMPS*co to yield vectors pRRL.PPT.nXC.2xBbsI.Cbx-SF.tbs.mTagBFP3-IRES-ODC.pre∗.SIN-BCx and pRRL.PPT.nXC.2xBbsI.Cbx-SF.tbs.YFP-IRES-OPRT.pre∗.SIN-BCx, respectively. mTagBFP3 was derived from the blue fluorescent protein mTagBFP2 with the same emission and excitation spectra. Next, a GeneArt String DNA fragment (Thermo Fisher Scientific) was ordered containing the sequence information for SpyTag003 and SpyCatcher003 and subcloned into pJet for sequence verification.^42^ Subsequently, the SpyTag003 and SpyCatcher003 domains were excised and ligated into *ODC* and *OPRT* lentiviral vectors to yield pRRL.PPT.nXC.2xBbsI.Cbx-SF.tbs.mTag-BFP3-IRES-SpyTag-ODC.pre∗.SIN-BCx and pRRL.PPT.nXC.2xBbsI.Cbx-SF.tbs.YFP-IRES-OPRT-SpyCatcher.pre∗.SIN-BCx, respectively.

FGB-ODC/OPRT split vectors (pRRL.PPT.nXC.2xBbsI.Cbx-SF.tbs.xFP-IRES-SpyTag-ODCco.pre∗xBC and pRRL.PPT.nXC.2xBbsI.Cbx-SF.tbs.CAAR-EGFRt-E2A-OPRT-SpyCatcher.pre∗SIN-BCx) were constructed based on the FGB-(U) vectors by exchanging the CAAR-E2A-UMPS cassette to IRES-SpyTag-ODCco and by excising the xFP and switching the *UMPS* to *OPRT*-SpyCatcher, respectively.

For the cloning of lentiviral vectors coexpressing antibiotic resistance genes, PCR products encoding puromycin N-acetyltransferase (Pac), blasticidin S deaminase (Bsd), and neomycin phosphotransferase II (Neo) were amplified from existing constructs using primer sets introducing 5′ NcoI and 3′ SalI compatible overhangs after digestion with BbsI or Esp3I. The resulting fragments—BbsI(NcoI)-Pac-(SalI)BbsI, Esp3I(NcoI)-Bsd-(SalI)Esp3I, and BbsI(NcoI)-Neo-(SalI)BbsI—were inserted into the pRRL.PPT.nXC.2xBbsI.Cbx-SF.tbs.hmAG3-P2A-CAAR-EGFRt-E2A-hUMPSco.pre∗.SIN-51BC backbone by three-fragment ligation. For this, the endogenous h*UMPS*co sequence was removed using KpnI in combination with NcoI and SalI, respectively, and replaced by the corresponding drug-resistance cassettes.

For the cloning of dLNGFR-expressing 51BC vectors, a synthetic 5′-NsiI-dLNGFR-2A-NcoI-3′ fragment (GeneArt String, Thermo Fisher Scientific) was used to replace EGFRt in the pRRL.PPT.nXC.2xBbsI.Cbx-SF.tbs.hmAG3-P2A-CAAR-EGFRt-E2A-hUMPSco.pre∗.SIN-51BC backbone. For this replacement, the vector was digested with NsiI and SalI, as well as NcoI and SalI, thereby excising the EGFRt cassette and enabling insertion of the dLNGFR-2A fragment.

To generate vectors co-expressing dLNGFR and antibiotic-resistance markers, a 2.3 kb endogenous dLNGFR fragment was excised by Esp3I digestion and ligated into the Esp3I-digested Pac, Bsd, or Neo versions of the pRRL.PPT.nXC.2xBbsI.Cbx-SF.tbs.hmAG3-P2A-CAAR-EGFRt-E2A backbone, in which the original hUMPSco cassette had already been replaced. PCR products and the GeneArt String were verified by Sanger sequencing. All restriction enzymes were purchased from New England Biolabs (NEB).

Additional cloning details are available on request.

### Lentiviral vector production

293T were cultured in DMEM (Gibco) supplemented with 10% heat-inactivated FBS (Sera Plus or FBS Standard, PAN-Biotech), 1% sodium pyruvate (Gibco), and 1% P/S (Gibco). For vector production, 9 μg lentiviral transfer vector, 13.3 μg psPAX2, and 2.7 μg pMD2.G (VSVg) plasmid were used for calcium phosphate mediated transfection of 10-cm cell culture dishes. The medium was changed 12–16 h post transfection, and supernatants were harvested 24 h and optionally 48 h later for ultracentrifugation, PEG-mediated precipitation or direct use. Aliquots were stored at −80°C until use.

### Transduction

5 × 10^4^ cells (H9M, THP1, or K562) were seeded into 96-well round bottom plates in 100 μL culture medium supplemented with 1 mg/mL Synperonic F108 (Sigma-Aldrich, St. Louis, Missouri, USA) (only H9M and THP1) and 4 μg/mL protamine sulfate (Sigma-Aldrich). After the addition of viral supernatants, the cells were placed into a humidified incubator at 37°C and 5% CO_2_. 6–16 h (48 h for K562 cells) post-transduction, the media was replaced with the appropriate cell culture media. Transduction rates were determined by flow cytometry 4–7 days after transduction. T cell cultures were transduced with 8 × 10^4^ cells/48 well plate in a total volume of 300 μL supplemented with 4 μg/mL protamine sulfate. The cells received fresh medium 36–48 h later.

### UMPS knockout

All gRNA sequences are listed in Table S1 sgRNAs were designed using CCTop (https://cctop.cos.uni-heidelberg.de/)^57^ and the CRISPRater score.^58^ The sequences were modified with respective overhangs (FW: 5′-CACCG, RV: 5′-AAAC) and obtained as oligonucleotides (Integrated DNA Technologies, Inc. (IDT), Coralville, USA). crRNAs were selected and obtained from IDT. UMPS-7 has been previously described.^28^

### Nucleofection

The nucleofection protocol is based on the user protocol “Electroporation of primary human CD34^+^ hematopoietic stem and progenitor cells” by Hendel et al*.*^59^ In short, 1.25 μL of each crRNA were mixed and incubated with 2.5 μL fluorescent tracrRNA (Atto). Ribonucleoparticles (RNPs) were generated by mixing and incubation of 1.2 μL crRNA:tracrRNA duplex with 1.7 μL Cas9 Nuclease V3 (62 μM). Up to 1 × 10^6^ cells/nucleofection well were washed twice with PBS, resuspended in 20 μL supplemented P3 Primary Cell Solution (Lonza; Basel, Switzerland) and mixed with 5 μL RNP complex and 1 μL electroporation enhancer (IDT). Electroporation was performed in a 16-well nucleocuvette using the 4D-Nucelofector (Lonza) and protocol EO-100 (cell lines) or EH-100 (T cells). Cells were resuspended in pre-warmed culture medium, incubated at 37°C for 20 min and then transferred onto a cell culture plate containing 900 μL culture media supplemented with 250 μg/mL uridine. Nucleofection rate was determined after 24 h by flow cytometry and detection of Atto-positive cells. Knockout efficiency was analyzed 48–96 h post-nucleofection by direct PCR, followed by sequencing and TIDE analysis (http://shinyapps.datacurators.nl/tide/).^29^

### PCR of *UMPS* locus from genomic DNA

All primers used for PCR were produced by IDT and are listed in Table S2. PCR reaction was set up using Phire Tissue Direct PCR Master Mix (Thermo Fisher Scientific) according to the manufactures instructions with annealing temperatures indicated in Table S2.

### Flow cytometric analysis

Flow cytometric analyses were performed on a spectral 3 laser (V-B-R) *Cytek Northern Lights*. Cells were washed, optionally stained for surface markers, and resuspended in FACS buffer (PBS, 2% [v/v] FBS and 4 mM EDTA) supplemented with 0.2 μg/mL 4′,6-diamidino-2-phenylindole (DAPI) if no fixable viability dye was used. Staining was performed in 50–100 μL FACS buffer using antibodies listed in Table S3. For analysis, cells were first gated by SSC-A vs. FSC-A, prior to single cell discrimination via FSC-H vs. FSC-A, and FSC-A vs. DAPI or Zombie for live/dead cell discrimination.

### Selection of UMPS-knockout cells

5-fluoroorotic acid (5-FOA, dissolved to 100 mg/mL in DMSO; Zymo Research, Irvine, California, USA) was directly added to each cell culture well. For the continuous treatment, cells were cultured in media containing 5-FOA for 1 week, while for the one-time treatment 5-FOA was removed after 2 days. 5-FOA was titrated and 10 μg/mL was determined as a standard for cell lines. T cells were treated with 5 μg/mL 5-FOA, instead. If necessary, 5-FOA treatment was repeated until all control cells had died.

### Antibiotic selection

4–7 days after transduction, gene marking rates of K562 WT and K562 *UMPS*^ko^ cells were determined by flow cytometry. Afterward, each well was split into two samples, of which one was left untreated and the other was exposed to 1 μg/mL puromycin, 10 μg/mL blasticidin and 750 μg/mL G418 (all InvivoGen), respectively. In parallel, K562 *UMPS*^ko^ cells were either subcultured with or without 250 μg/mL uridine. Antibiotic treatment was stopped after 7 days, after which gene marking in all samples was assessed by flow cytometry in weekly intervals.

### Western blotting

Protein extracts from wild-type cells, *UMPS^ko^* cells, and transduced *UMPS^ko^* cells were prepared using RIPA buffer. Equivalent protein amounts were resolved by SDS-PAGE and transferred onto PVDF membranes (Amersham). Following blocking with 5% skim milk, UMPS was detected using a mouse anti-human primary antibody (clone A-9; Santa Cruz Biotechnology), and an HRP-conjugated goat anti-rat secondary antibody (clone #Poly4054; BioLegend). GAPDH was likewise detected by a monoclonal mouse IgG_1_ (clone #686613, R&D Systems) and peroxidase-conjugated polyclonal secondary goat anti-mouse IgG (# 610–1302; Rockland).

### Statistical analysis

Population sizes were quantified by flow cytometry and are reported as mean ± SD from *n* independent biological replicates as indicated in each figure. Statistical differences between two groups were calculated using the Mann-Whitney U test. Statistical analyses between multiple groups were assessed using the Kruskal-Wallis test followed by Dunn’s multiple-comparison correction and two-way analysis of variance (ANOVA) with Dunnett multiple comparisons correction, respectively, as indicated in the figure legends. A *p* value of ≤0.05 was considered statistically significant (∗*p* ≤ 0.05, ∗∗*p* ≤ 0.01, ∗∗∗*p* ≤ 0.001, ∗∗∗∗*p* ≤ 0.0001). All statistical analyses were performed using GraphPad Prism.

## Data availability

All data are included in the manuscript. Graphics presented in the figures were created in BioRender (Steding, H. (2026); https://BioRender.com/8hd1qux).

## Acknowledgments

We are grateful to Florian Kuchenbauer and all members of his lab for the scientific support and helpful discussions. We appreciate the support provided by Axel Schambach (Hannover Medical School) for helpful discussions and for sharing vector constructs. We are also grateful to Katharina Zimmermann for advice regarding T cell cultures. Parts of this work were included in the dissertation of Henrike Steding (2024 Hannover Medical School). This project was partly funded through the “Verein für krebskranke Kinder Hannover e.V.” and by an add-on fellowship of the Joachim Herz Stiftung to HS.

## Author contributions

Conceptualization, T.M.; methodology, T.M. and H.S.; investigations, H.S., N.D., J.H., and T.M.; resources, T.M. and M.S.; writing the original draft, H.S. and T.M.; writing-review and editing, T.M., M.S., and H.S.; visualization, H.S. and T.M.; supervision, T.M.; funding acquisition, T.M.

## Declaration of interests

The authors declare no competing interests.

## Supplemental information

Document S1. Figures S1–S6 and Tables S1–S3

Document S2. Article plus supplemental information
