# Supplementary material for: Lentiviral rescue of UMPS auxotrophy enables drug-free selection and stable vector expression
Source: Mol Ther Adv. 2026 May 15;34(2):201761. doi: 10.1016/j.omta.2026.201761 (PMC13254677; doi:10.1016/j.omta.2026.201761)
Supplement: Document S1. Figures S1–S6 and Tables S1–S3 [file mmc1.pdf]

OMTA, Volume *34*

Supplemental information

Lentiviral rescue of UMPS auxotrophy enables drug-free selection and stable vector expression

Henrike Steding, Nicole Dörpmund, Jessica Herbst, Martin Sauer, and Tobias Maetzig

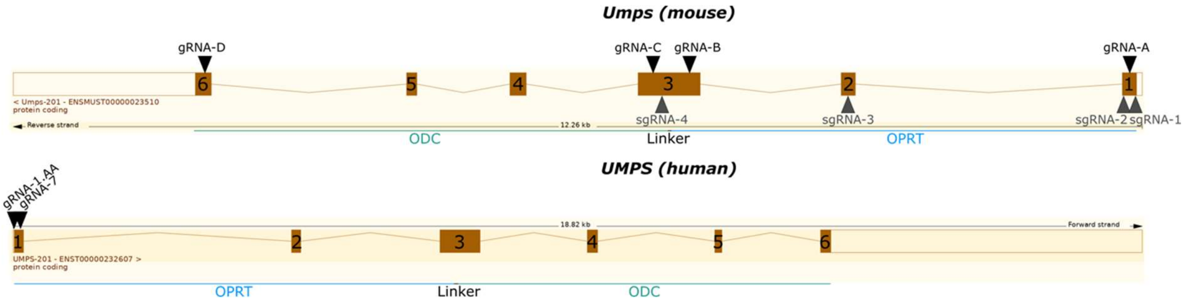

Figure S1: Location of sgRNAs relative to the mouse and human UMPS gene. Top: Murine *Umps* gene and approximate sgRNA (-1, -2, -3, -4) and crRNA (-A, -B, -C, -D) target sites. Bottom: Human *UMPS* gene and approximate crRNA target sites (UMPS-7, UMPS-1.AA). Exons are shown as brown boxes and numbered. OPRT (exon 1-3) and ODC (exon 3-6) subunits are connected through a linker region in exon 3.

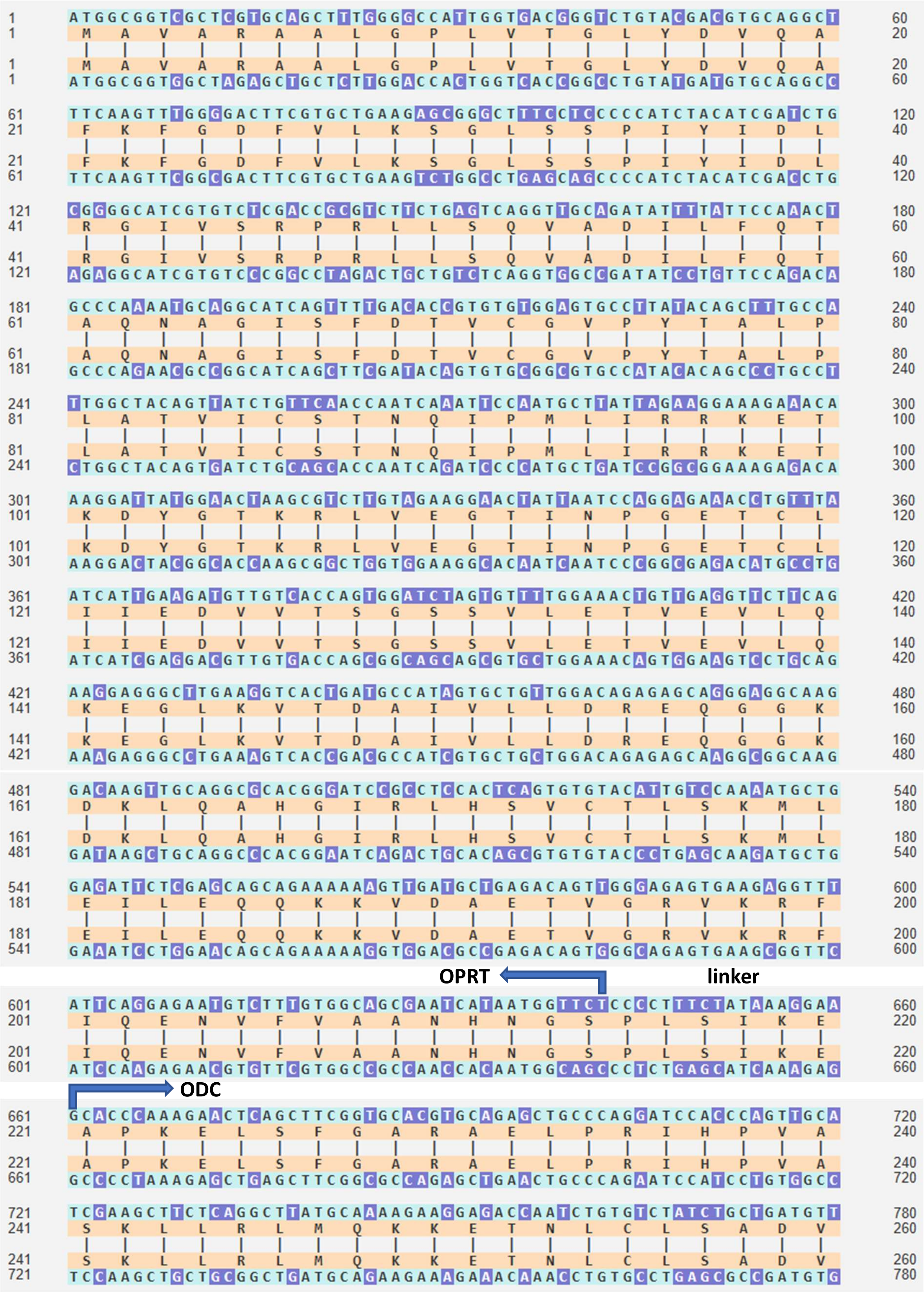
OPRT
linker
ODC

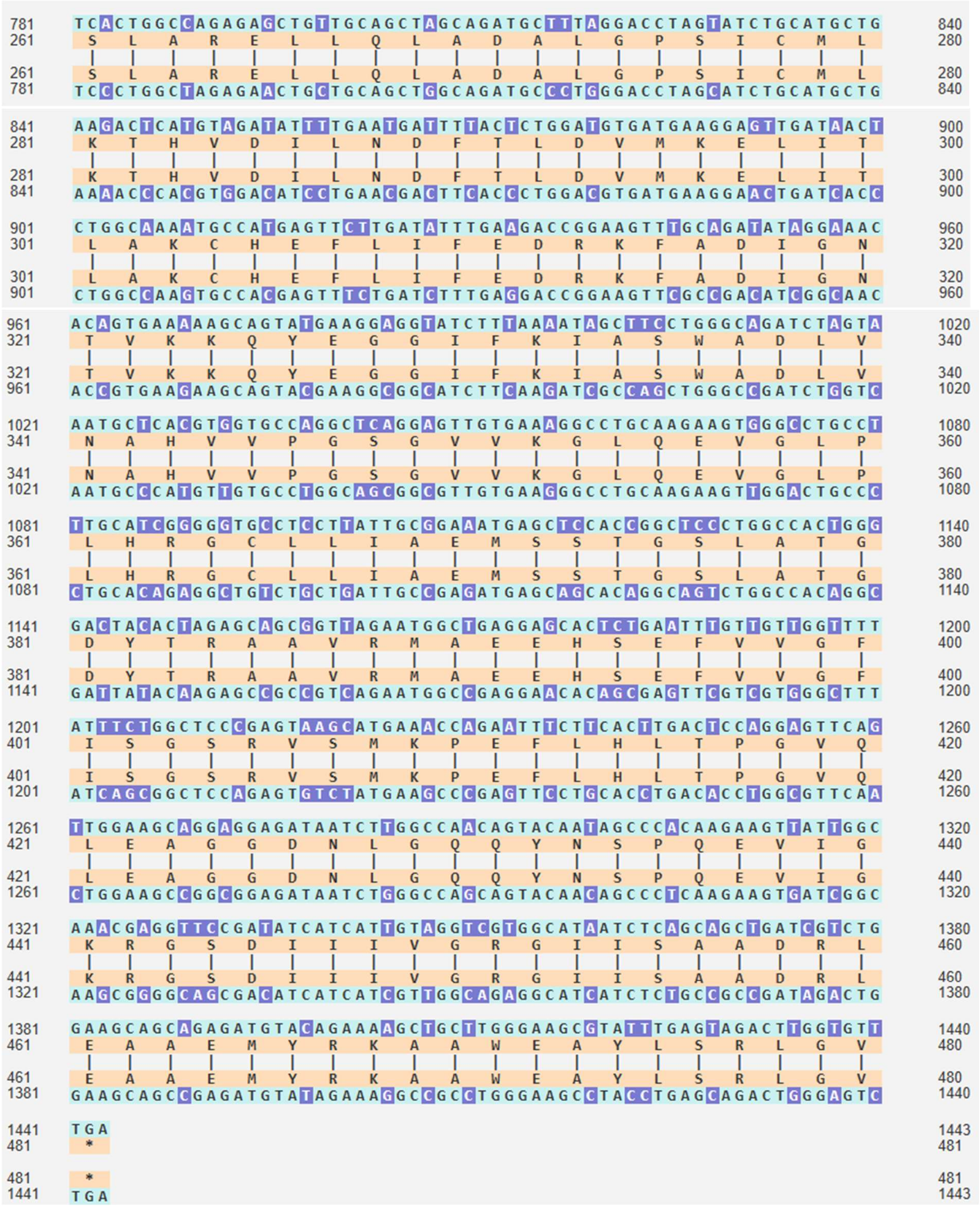

Figure S2: Alignment of the wild-type and codon-optimized human UMPS cDNA sequence. The alignment was done with the VectorBuilder alignment tool and displays the wild-type sequence with its nucleotide and amino acid sequence at the top, and the corresponding information of the codon-optimized h*UMPS*co at the bottom. Mismatching nucleotides are displayed in purple. Domains for OPRT (nt 1 - 642) and ODC (nt 661 – 1440) are indicated by arrows. CCDS3029.1 was used as *UMPS* reference sequence.

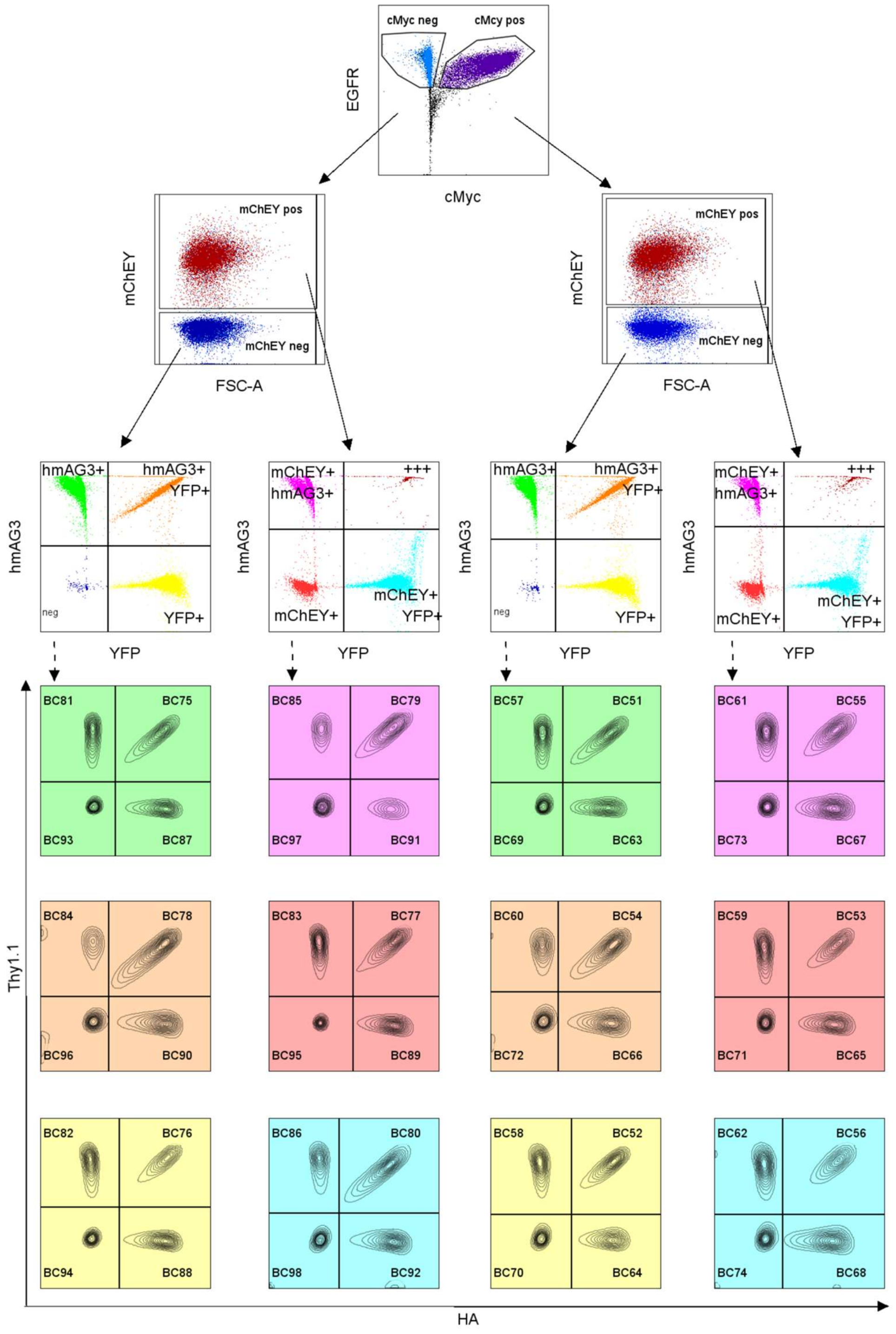

Figure S3: Flow cytometric deconvolution of all 48 input color codes of the 48x FGB vector series. The following gating strategy was applied: Transduced cells are identified by EGFRt expression and divided in 2 sublibraries (+/- cMyc). Next, xFP expression is determined by plotting mChEY vs FSC-A, followed by hmAG3 vs YFP. Finally, all populations expressing either one or two xFP are then plotted for Thy1.1 vs HA expression, which results in a total of 48 subpopulations.

A

```
agacagtggg cagagtgaag cggttcatcc aagagaacgt gttcgtggcc gccaaccaca atggcagcgg ttccggtggc tcgggtggat ctatggtgac
tctgtcaccc gtctcacttc gccaagtagg ttctcttgca caagcaccgg cggttggtgt taccgtcgcc aaggccaccg agcccaccta gataccactg
                                                                                             c to t silent mutation
 e  t  v   g  r  v  k   r  f  i   q  e  n   v  f  v  a   a  n  h   n  g  s   g  s  g  g   s  g  g   s  m  v
>...............................hOPRTco...............................>>
 e  t  v   g  r  v  k   r  f  i   q  e  n   v  f  v  a   a  n  h   n  g  s
                                                                                           SpyCatcher003 >>.....>
                                                                                                         m  v

cacactgtcc ggactgtctg gagagcaggg accatccggc gacatgacca cagaggagga ttctgccaca cacatcaagt tcagcaagag ggacgaggac
gtgtgacagg cctgacagac ctctcgtccc tggtaggccg ctgtactggt gtctcctcct aagacggtgt gtgtagttca agtcgttctc cctgctcctg
t  t  l  s   g  l  s   g  e  q   g  p  s  g   d  m  t   t  e  e   d  s  a  t   h  i  k   f  s  k   r  d  e  d
>...........................................SpyCatcher003...........................................>
t  t  l  s   g  l  s   g  e  q   g  p  s  g   d  m  t   t  e  e   d  s  a  t   h  i  k   f  s  k   r  d  e  d

ggaagagagc tggcaggagc aacaatggag ctgagggata gctccggcaa gaccatcagc acatggatct ccgacggcca cgtgaaggat ttctacctgt
ccttctctcg accgtcctcg ttgttacctc gactccctat cgaggccgtt ctggtagtcg tgtacctaga ggctgccggt gcacttccta aagatggaca
                c to a silent mutation
  g  r  e   l  a  g   a  t  m  e   l  r  d   s  s  g   k  t  i  s   t  w  i   s  d  g   h  v  k  d   f  y  l
>...........................................SpyCatcher003...........................................>
  g  r  e   l  a  g   a  t  m  e   l  r  d   s  s  g   k  t  i  s   t  w  i   s  d  g   h  v  k  d   f  y  l

atcccggcaa gtacaccttt gtggagacag cagcaccaga cggatatgag gtggcaaccc ctatcgagtt tacagtgaac gaggacggac aggtgaccgt
tagggccgtt catgtggaaa cacctctgtc gtcgtggtct gcctatactc caccgttggg gatagctcaa atgtcacttg ctcctgcctg tccactggca
 y  p  g   k  y  t  f   v  e  t   a  a  p   d  g  y  e   v  a  t   p  i  e   f  t  v  n   e  d  g   q  v  t
>...........................................SpyCatcher003...........................................>
 y  p  g   k  y  t  f   v  e  t   a  a  p   d  g  y  e   v  a  t   p  i  e   f  t  v  n   e  d  g   q  v  t

                                                            SalI
ggatggagag gcaacagagg gcgatgcaca caccggatct agcggatctt aggtcgacat tatggagcat cttaccgcca tttataccca tatttgttct
cctacctctc cgttgtctcc cgctacgtgt gtggcctaga tcgcctagaa tccagctgta atacctcgta gaatggcggt aaatatgggt ataaacaaga
v  d  g  e   a  t  e   g  d  a   h  t  g  s   s  g  s   -  v  d   i  m  e  h   l  t  a   i  y  t   h  i  c  s
>...................SpyCatcher003...................>>
v  d  g  e   a  t  e   g  d  a   h  t  g  s   s  g  s   -
```

B

```
caaccatggg ccgtggcgtg cctcatatcg tgatggtgga cgcctacaag cgttacaagg ccggtccgat ggcccctaaa
gttggtaccc ggcaccgcac ggagtatagc actaccacct gcggatgttc gcaatgttcc ggccaggcta ccggggattt
 t  t  m   g  r  g  v   p  h  i   v  m  v   d  a  y  k   r  y  k   a  g  p   m  a  p  k
        >>....................SpyTag003.....................>>
           g  r  g  v   p  h  i   v  m  v   d  a  y  k   r  y  k
                                                                            >>.hODCco...>
                                                                               m  a  p  k
```

Figure S4: Nucleotide and amino acid sequence of SpyCatcher / SpyTag UMPS subunits. A. 3'sequence of hOPRTco fused to SpyCatcher003. Silent point mutations have been introduced into SpyCatcher003 to destroy NcoI sites. **B.** 5' SpyTag003 fused to hODCco. Complete h*UMPS*co sequence is shown in Figure S2.

A B C

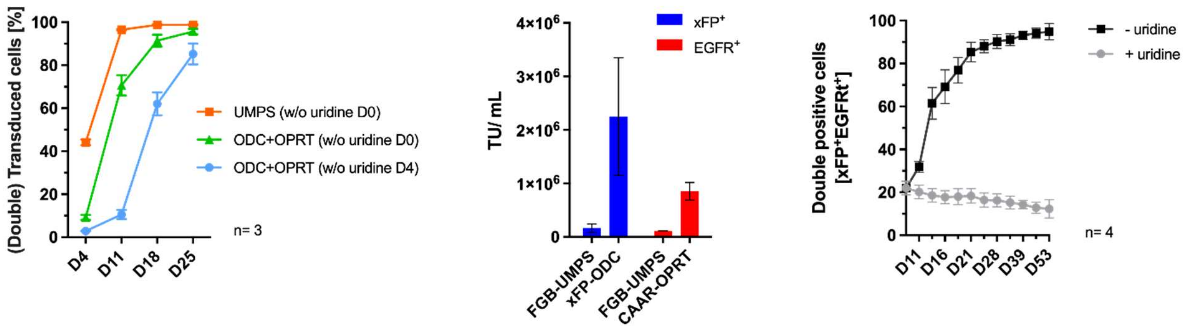

Figure S5: Influence on titer and selection dynamics of the split-UMPS vector design. A. Comparison of enrichment by monocistronic and split-UMPS constructs. THP1 *UMPS*^{ko} cells were transduced with eGFP-UMPS or co-transduced with mTagBFP3-Spy-ODC and YFP-OPRT-Spy. Transduction was performed either in the absence of uridine (w/o uridine D0) or uridine withdrawal was started 4 days after transduction (w/o uridine D4). Mean of n= 3. **B.** Comparison of viral titer between the FGB-UMPS vector and the xFP-ODC or CAAR-OPRT vector. Transduction units [TU]/ mL were calculated based on detection of either fluorescent protein (xFP$^+$) or stained EGFRt (EGFRt$^+$) by flow cytometry. Mean of 48 individual transductions pooled and measured in 3 replicates (FGB-UMPS) and mean of 144 individual co-transductions measured individually (xFP-ODC and CAAR-OPRT). **C.** Enrichment of double transduced cells (xFP$^+$EGFRt$^+$) in the absence of uridine (starting on day 4 after transduction) in samples with high transduction rate. In contrast, uridine supplementation prevents the expansion of xFP$^+$EGFRt$^+$ cells. Mean of n= 4 different vector combinations. Bars indicate mean ± SD.

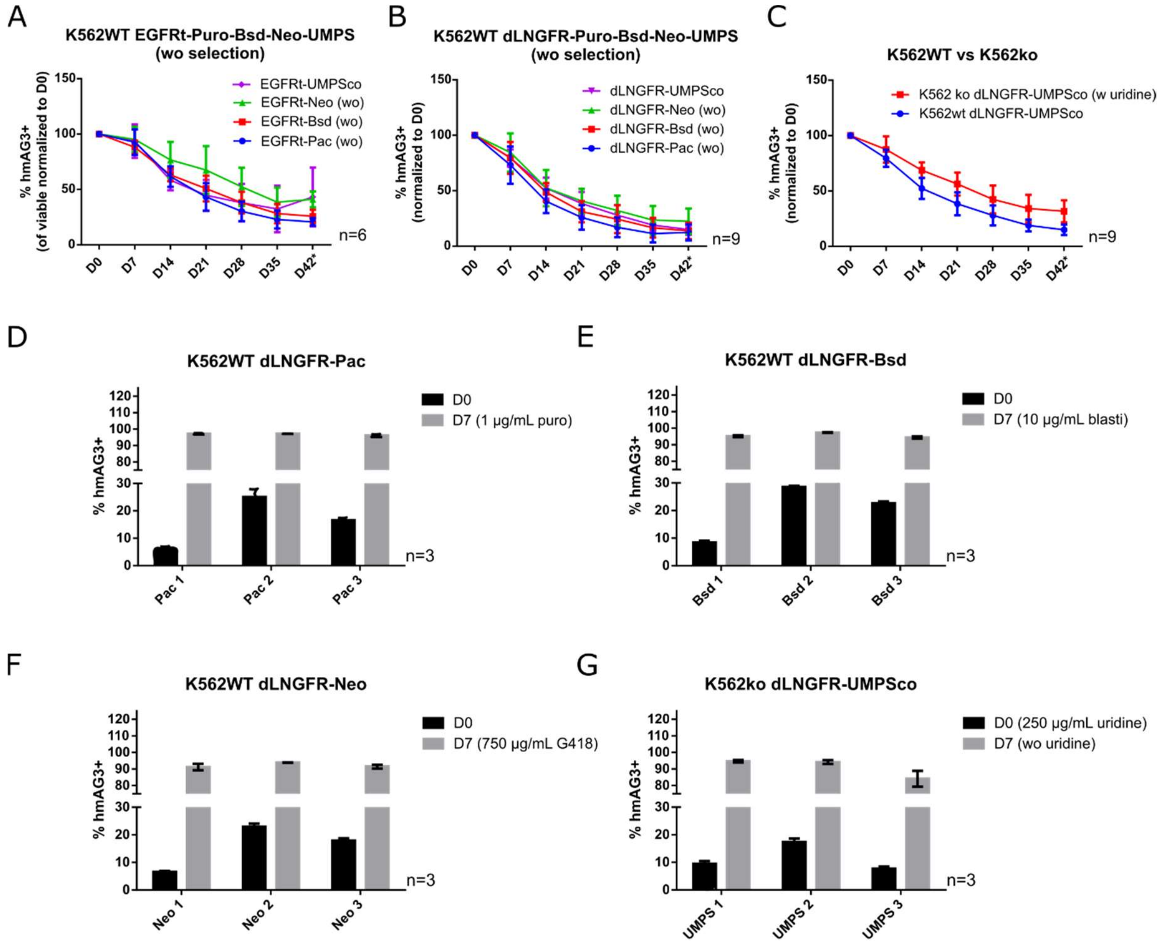

Figure S6: Drug- and UMPS-mediated selection efficiencies in K562WT and K562 *UMPS*^{ko} cells. A-B. Normalized gene marking rates in K562 cells transduced with vectors coexpressing **A.** EGFRt or **B.** dLNGFR and antibiotic-resistance genes (Pac, Bsd, and Neo) or h*UMPS*co. Vector constructs are shown in Figure 6A. Data have been used to generate Figure 6D-E. **C.** Comparison of gene marking rates between K562 wild-type (WT) and K562 *UMPS*^{ko} (ko) cells transduced with dLNGFR vectors coexpressing h*UMPS*co. K562 *UMPS*^{ko} cells were cultivated with uridine. Measurements on day D42* in A-C were only done with n=3 (EGFRt constructs) and n=6 (dLNGFR constructs). **D-F.** Antibiotic treatment of transduced K562 cells occurred between day 0 and day 7, and flow cytometric analyses were performed on both days to assess the gene marking rate and cell enrichment after one week of selection. **G.** Selection efficiency of transduced K562 *UMPS*^{ko} cells before and 7 days after withdrawal of uridine. The data from this graph have also been used to generate Figure 3C. The data in **D-G** were generated from three independent experiments performed in triplicates. Bars indicate mean ± SD.

Table S1: sgRNAs and crRNAs

Name	Target gene	Species	crRNA sequence
crRNA-UMPS.1_T4 (sg24+25)	Umps	Mouse	AACTGAGCGCGCGAGAATGG
crRNA-UMPS.1_T7 (sg26+27)	Umps	Mouse	AGTCTACATCGACCTGCGGG
crRNA-UMPS.2_T3 (sg28+29)	Umps	Mouse	ACATTCCCATGCTCATTAGG
crRNA-UMPS.3_T1 (sg30+31)	Umps	Mouse	TCTGTCTGCCGATGTGTCGG
Mm.Cas9.UMPS.1.AA (A)	Umps	Mouse	CACCGAGCTGTATGACGTGC
Mm.Cas9.UMPS.1.AB (B)	Umps	Mouse	AAGATCGACGCTGACATGGT
CD.Cas9.LCVV4042.AA (C)	Umps	Mouse	GAACTCGTGGCGTTTTGCCA
CD.Cas9.VWRD2185.AA (D)	Umps	Mouse	CAAGAAGTGATCGGGAAACG
UMPS-7	UMPS	Human	GCCCCGCAGATCGATGTAGA
Hs.Cas9.UMPS.1.AA	UMPS	Human	TCGTACAGACCCGTCACCAA

Table S2: UMPS locus PCR primer

Name	Sequence	Covering cut side of	Species	Annealing Temp.
HS_UMPS.1_T4_FW	GTGGCTGTTAACAGTGACTGG	sgRNA_1	mouse	64 °C
HS_UMPS.1_T4/T7_RV*	TAATTTCTCCACCCTCCCGCC			
HS_UMPS.1_T7_FW	CGGGACTTCTGGGTGACGTCA	sgRNA_2		
HS_UMPS.1_T4/T7_RV*	TAATTTCTCCACCCTCCCGCC			
HS_UMPS.2_T3_FW	CTGTTGTGCTGTGGCATGATTC	sgRNA_3		
HS_UMPS.2_T3_RV	AAGCAAACACACCTGACGTGG			
HS_UMPS.3_T1_FW	TGGAGATTCTCCAGCAGCAGG	sgRNA_4		
HS_UMPS.3_T1_RV	GACAGGATTCTGAGTCCTCGA			
HS_hUMPS_FW	CCCGGGGAAACCCACGGGTGC	UMPS-7 and Hs.Cas9. UMPS.1.AA	human	70 °C
HS_hUMPS_RV	AGGGTCGGTCTGCCTGCTTGGCT			

Table S3: FGB antibody panel

Antibody	Labeled with	Company, product number	Dilution
cMyc	Biotin	Miltenyi, 130-124-899	1:50
Streptavidin	BV605	Biolegend, 405229	1:100
EGFR	APC	Biolegend, 352906	1:400
Thy1.1	PE-Cy7	Biolegend, 202517	1:1000- 1:2500
HA	PE	Miltenyi, 130-120-717	1:100
